# A 10 year study of the cause of death in children under 15 years in Manhiça, Mozambique

**DOI:** 10.1186/1471-2458-9-67

**Published:** 2009-02-24

**Authors:** Jahit Sacarlal, Ariel Q Nhacolo, Betuel Sigaúque, Delino A Nhalungo, Fatima Abacassamo, Charfudin N Sacoor, Pedro Aide, Sonia Machevo, Tacilta Nhampossa, Eusébio V Macete, Quique Bassat, Catarina David, Azucena Bardají, Emili Letang, Francisco Saúte, John J Aponte, Ricardo Thompson, Pedro L Alonso

**Affiliations:** 1 Centro de Investigação em Saúde da Manhiça (CISM), Mozambique; 2 Faculdade de Medicina, Universidade Eduardo Mondlane, Maputo, Mozambique; 3 Barcelona Centre for International Health Research (CRESIB), Hospital Clínic/IDIBAPS, Universitat de Barcelona, Barcelona, Spain; 4 National Directorate of Health, Ministry of Health, Maputo, Mozambique; 5 National Institute of Health, Ministry of Health, Maputo, Mozambique

## Abstract

**Background:**

Approximately 46 million of the estimated 60 million deaths that occur in the world each year take place in developing countries. Further, this mortality is highest in Sub-Saharan Africa, although causes of mortality in this region are not well documented. The objective of this study is to describe the most frequent causes of mortality in children under 15 years of age in the demographic surveillance area of the Manhiça Health Research Centre, between 1997 and 2006, using the verbal autopsy tool.

**Methods:**

Verbal autopsy interviews for causes of death in children began in 1997. Each questionnaire was reviewed independently by three physicians with experience in tropical paediatrics, who assigned the cause of death according to the International Classification of Diseases (ICD-10). Each medical doctor attributed a minimum of one and a maximum of 2 causes. A final diagnosis is reached when at least two physicians agreed on the cause of death.

**Results:**

From January 1997 to December 2006, 568499 person-year at risk (pyrs) and 10037 deaths were recorded in the Manhiça DSS. 3730 deaths with 246658 pyrs were recorded for children under 15 years of age. Verbal autopsy interviews were conducted on 3002 (80.4%) of these deaths. 73.6% of deaths were attributed to communicable diseases, non-communicable diseases accounted for 9.5% of the defined causes of death, and injuries for 3.9% of causes of deaths. Malaria was the single largest cause, accounting for 21.8% of cases. Pneumonia with 9.8% was the second leading cause of death, followed by HIV/AIDS (8.3%) and diarrhoeal diseases with 8%.

**Conclusion:**

The results of this study stand out the big challenges that lie ahead in the fight against infectious diseases in the study area. The pattern of childhood mortality in Manhiça area is typical of developing countries where malaria, pneumonia and HIV/AIDS are important causes of death.

## Background

One of the eight Millennium Development Goals (MDG), aims to reduce under five mortality rates by two-thirds, especially in African countries. To accomplish this goal, researchers, programme planners and policy-makers need information on the causes of death occurring in countries [1]. Approximately 46 million of the estimated 60 million deaths that occur in the world each year take place in developing countries [2]. Further, this mortality is highest in Sub-Saharan Africa, although causes of mortality in this region are not well documented [3]. In order to most appropriately allocate resources and to evaluate the impact of individual illnesses and its control, it is important to know the number and proportion of deaths in a community likely to be due to a given condition. In rural areas of developing African countries such as Mozambique, where many children are born and die without ever being registered, and where a significant proportion of all deaths take place outside health facilities, the only way of estimating the likely cause of death is through an interview of a witness of final illness. This is called a verbal autopsy (VA) [3-6].

## Methods

### Study area

Manhiça district is located in southern Mozambique, in the Maputo Province, about 80 km north of Maputo City. The area has two distinct regions. The first is the fertile lowlands, comprising the Incomati River flood plain running from the northern to the southern district boundary. This area is poorly inhabited and used mainly for sugarcane and fruit plantations. The second area is an escarpment of moderate altitude bordering the west of the river, where the population inhabits an extensive plateau. There are two distinct seasons, a warm and rainy season between November and April and dry and cool season during rest of the year. A full description of the geographical and sociodemographic characteristics of the study area has been presented elsewhere [7,8].

The Manhiça Demographic Surveillance System (DSS) in Manhiça District was established in 1996, and currently covers a 500 square kilometre area. An initial census was carried out in 1996, and vital events registration (births, deaths, pregnancy and, in/out-migration) were conducted on quarterly basis until the year 2000, when this was changed to twice yearly.

Verbal Autopsies (VA) data collection started in 1997 with the aim of generating cause-specific mortality data in the study area. Initially, VA were conducted only in January and July on deaths of children aged less than 15 years reported through the DSS in the previous 6 months. Since the introduction of new questionnaires in June 2002 through the MTIMBA (Malaria Transmission Intensity and Mortality Burden Across Africa) project from INDEPTH, VA interviews are carried out every day by a well-trained lay supervisor and field workers.

### Description of the health delivery system

There are two referral health facilities in Manhiça district, the Manhiça District Hospital (MDH), with 110 beds, and the Xinavane Rural Hospital (XRH), with 59 beds. In addition, 10 peripheral health facilities complete the official health facilities network. Most of the government medical services are provided free of charge except for drugs prescribed at the outpatient department that is available for purchase at subsidized prices. Adults pay a symbolic consultation fee of about USD 0.02.

Since 1996 the Manhiça Health Research Center (CISM) has been operating a round-the-clock, hospital-based morbidity surveillance system for children under 15 years of age attending the MDH and three other peripheral health facilities in the study area [8].

Voluntary counselling and testing to prevent mother to child transmission with Niverapina since 2003, and Highly Active Anti Retroviral Therapy (HAART) are available since 2004 for all patients including pregnant women in MDH, according to national policies.

Obstetric services including obstetric emergency care, operation room and morbidity surveillance system were established at the MDH maternity clinic, as a passive case detection system, for all women (pregnant, puerperal and women with gynaecological complaints) attending this clinic with clinical complaints (i.e., not for those attending the routine antenatal clinic).

### Selected indicators for the DSS

Between 1997 and 2005, the number of inhabitants living in the study area increased from 32856 to 79783, due to population growth and the extension of the DSS area in August 2002. During these years, the total fertility rate decreased from 5.2 to 4.8 children per woman. The infant mortality risk in 2005 was 77.5 per 1000 live births, the under five mortality (5q0) rate was 138.6 deaths per 1000 pyrs, and the life expectancy at birth was 40.2 years [9].

### Data collection & processing

The methodology used in identifying vital events in the study area has been fully described elsewhere [7]. Information on deaths comes from one of several sources: (I) household visits twice a year that are conducted to record all deaths and other demographic events that have occurred since the previous visit, (II) daily visits to hospital wards and maternity clinics by supervisors to gather information on all deaths and pregnancy related events that have taken place in the previous 24 hours and (III) weekly reports by local key informants on births, deaths and migrations that might be missed during household census visits by field workers and supervisors. Age is ascertained by direct questioning, referral to any existing personal identification documents and, if necessary, an area-specific calendar of events is used. An identification card is issued to all children under 15 years of age to allow identification of patients in the morbidity surveillance system in the MDH.

Initially, eight medical students conducted VA interviews in the study area twice a year. The work was supplemented after June 2002 by a lay supervisor and field workers who interviewed key community informants and relatives of the deceased, daily. Between three and six months after a death, a field worker visited the family of the deceased to inquire whether they would accept to participate in a verbal autopsy. Upon acceptance, an oral consent was obtained from the interviewee and a date for the interview was agreed. On the day of the interview, a signed or fingerprinted informed consent (IC) was sought before the VA took place. To ensure consent within the family, potential interviewers were given an information sheet with study objectives and procedures during the initial contact, and were encouraged to discuss with family members before proceeding. Interviewers who could not read were free to ask their relatives to read the document for them. The primary informant was, whenever possible, the person who directly took care of the deceased child during the illness or condition that led to death. If the primary respondent was absent, information was sought from any other adult, including neighbours, who might have relevant information on the possible cause of death. In order to maintain confidentiality, only the coding physicians and the data entry clerks had access to the assigned causes of death.

A demographer was in charge of controlling the data quality through an on-site review of questionnaires. After fieldwork, all questionnaires were checked for consistency and completeness. Questionnaires needing corrections were returned to the field within two weeks of their receipt.

### Nature of the VA tool

The study used a VA questionnaire standardized from INDEPTH [7] and adapted from the WHO model [10]. The standard questionnaire in Manhiça was written in Portuguese. However, the fieldworkers perform an on-site translation of the questions into the local language (Xangana). The questionnaire included questions on the identification of the deceased and the respondent as well as the health seeking behaviour and use of health services by the deceased prior to the death. The questionnaire also had an open-ended section where circumstances surrounding the death of the child, as well as the signs and symptoms presented during the illness preceding death, are recorded. The final section had closed questions on signs and symptoms preceding the death that did not focus on any particular disease.

### Assigning the cause of death

To assign the cause(s) of death, diagnoses are given using a standardised coding system. Three physicians with experience in tropical diseases independently assigned the cause of death using the International Classification of Diseases (ICD-10) [11]. Each physician ascribes a minimum of one and a maximum of 2 causes. Conditions should be additive and not alternative. For example, if more than one diagnosis was mentioned, it may be classified as "malaria or pneumonia," but should be stated as "malaria and pneumonia". A final diagnosis was reached when at least two physicians agree on the cause of death. When at least two physicians assigned "unknown" as the cause of death, the final cause of death was considered undetermined. When the cause was different among the three reviewers, the final diagnosis was "not consensus", and these deaths were not redistributed to other diagnosis groups. When two final diagnoses were assigned for the same death, each of these was individually mapped onto ICD-10 for a calculated cause-specific rate.

To rank causes of death, we used the GBD tree structures [12]. The first level included three mortality groups: Group 1 consisted of deaths attributed to communicable diseases and to maternal, perinatal and nutritional conditions; Group 2 comprised deaths attributed to non-communicable diseases and, Group 3 comprised deaths due to injuries. Each of the three groups was further divided into several major subcategories (second to fourth level). Third and fourth levels were used to classify specific causes of death.

### Data Management & Analysis

Trained data entry clerks and a data manager ensured data entry into a network of computers under a Windows NT environment. Double data entry was performed by two clerks using a modified version of The Household Registration System (HRS) [13]. Inconsistencies, if any, were corrected after counter-checking with the original questionnaires. Questionnaires with errors that could not be reconciled were returned to the field for correction. The database with the VA data was linked to other DSS databases. Data management, cleaning and statistical analysis were performed using STATA (Stata Corporation 2005, Stata Statistical Software: Release 9.2 College Station, TX: StataCorp LP, USA).

### Calculation of mortality rates

Time at risk of disease was calculated for each individual registered in the demographic surveillance system, subtracting periods of absence due to migration. All-cause mortality rates were calculated by dividing the number of deaths in an age group by the time at risk, and expressed as deaths per 1000 person-years at risk. We calculated cause-specific death rates for each age group by multiplying the all-cause mortality rate by the proportion of deaths assigned to each cause.

### Ethical considerations

The study falls within the national ethical clearance granted to the malaria epidemiological studies of the CISM (Ministry of Health/National Institute of Health of Mozambique, 1996). The participation of the respondents during the interview was voluntary and conducted only after the IC procedure described earlier. The interviews were conducted at least one month after death, when the traditional grieving period was over.

## Results

### Population size and characteristics

From January 1997 to December 2006, 568499 person-year at risk (pyrs) and 10037 deaths were recorded in the Manhiça DSS. 3730 deaths with 246658 pyrs were recorded in children under 15 years old. Verbal autopsy interviews were conducted for 3002 (80.4%) of these deaths. Non-completion was due to family out-migration (9.8%), prolonged absence of the relatives of the deceased (3.9%) or refusals (3.2%). Forty seven percent of the interviews were conducted within a period of 6 months and 83.9% within 1 year from the time of death. The median time was 8 months. According to respondents, 54% of deaths occurred outside a health facility. However, medical and other assisted care during the terminal illness was sought by 81.9% of those who died. Sources of care included: health centers and hospitals (67.8%), traditional healers (8.3%), religious leaders (1.8%), friends and family (1.1%) and others (20.9%).

### Age and sex distribution of deaths

Most of the paediatric deaths occurred in children aged 1–4 years (41.3%), followed by infants aged 29 days – 1 year (30.6%). Overall, males constituted 54.7% of the total children analyzed (Table 1).

**Table 1:** Distribution of deaths and verbal autopsy by age and sex in Manhiça DSS, 1997–2006

	DSS*, MORTALITY				**Verbal Autopsy**					
		
**Age group**	**No deaths**	**%**	**pyar**	CDR**/1000 pyrs	**No Male**	**%**	**No Famale**	**%**	**No Total**	**%**
< 1 y	1757	47,1	21003	84	773	47	635	46,7	1308	43,6
1–4 y	1541	41,3	76343	20	671	40,8	568	41,8	1239	41,3
5–9 y	295	7,9	81729	4	136	8,3	103	7,6	239	7,9
10–14 y	137	3,7	67583	2	63	3,8	53	3,9	116	3,9
**Total**	**3730**		**246658**	**15**	**1643**		**1359**		**3002**	

*_DSS – Demographic Surveillance System

**_CDR-Crude Death Rate

### Main causes and mortality rate of registered death

Table 2 summarizes the distribution of 3696 causes by group for the 3002 deaths. Communicable diseases were responsible for most deaths (73.6%), non-communicable diseases accounted for 9.5%, and injuries for 3.9%. Among communicable disease, the most frequent diagnosis was infectious and parasitic diseases (60.0%), followed by perinatal disease (17.4%). Anaemia was a very common diagnosis with 54.8% of total causes of death among non-communicable diseases. Injury, poisoning drowning and certain other consequences of external causes were also common in group 3.

**Table 2:** Distribution of registered deaths by group and level-two cause in Manhiça DSS, Mozambique, 1997–2006.

**Cause of death**	**No of diagnosis**	**%**
**I. Communicable Disease**		
Infectious and parasitic disease	1604	58,9
Perinatal disorders	473	17,4
Respiratory infections	361	13,3
Nutritional disorders	240	8,8
Others	43	1,6
**Total**	**2721**	**73,6**

**II. Non-communicable Disease**		
Blood disease	193	54,8
Metabolic disorders	49	13,9
Congenital abnormalities	34	9,7
Neuropsychiatric disorders	28	8,0
Digestive disorders	12	3,4
Genitourinary disorders	11	3,1
Respiratory disorders	10	2,8
Cardiovascular disorders	9	2,6
Malignant disorders	3	0,9
Skin disorders	3	0,9
**Total**	**352**	**9,5**

**III. Injuries**		
Injury, poisoning and certain other consequences of external causes	86	58,5
External causes of morbidity and mortality	61	41,5
**Total**	**147**	**4,0**

**IV. Undetermined and badly defined symptoms**	**106**	**2,9**
		
**V. No consensus**	**370**	**10,0**

**Total**	**3696**	

The more frequent double death causes are malaria and anaemia with 13,5% (94/694) of cases followed by HIV/AIDS and anaemia with 12,1% (84/694) of cases, and malaria and diarrhoea disease with 9,8% (68/694) of cases.

Table 3 shows the twenty leading causes of death and specific crude death rate for different causes as reported from VA for children less than 15 years of age. Malaria was the leading cause, accounted for 21.8% of total diagnosis given physicians and for 3.2 deaths/1000 pyrs. Acute lower respiratory infection (ALRI) including pneumonia, was the second leading cause of death with 9.8% of total diagnosis and 1.5 deaths/1000 pyrs, followed by HIV/AIDS with 8.3% and 1.3 deaths/1000 pyrs. Diarrheal diseases with 8.0% and 1.2 deaths/1000 pyrs and malnutrition with 6.4% and 0.96 deaths/1000 pyrs were other important conditions.

**Table 3:** The 20 leading causes of verbal autopsy deaths and mortality rate by sex and relative risk in Manhiça DSS, Mozambique, 1997–2006

	**Cause of death (block-ICD 10)**	**No of diagnosis**	**%**	**CDR Male**	**CDR Female**	**CDR Total**	**RR all age**	**pvalue**	**95% IC**
1	Malaria (B50-B54)	805	21,8	3,4	3,1	3,2	1,07	0,367	0,93–1,22
2	ALRI (Pneumonia) (J10-J18)	361	9,8	1,5	1,4	1,5	1,08	0,494	0,87–1,32
3	HIV/AIDS (B20-B24)	307	8,3	1,3	1,2	1,2	0,99	0,953	0,79–1,24
4	Diarrhoeal diseases (A00-A09)	297	8,0	1,3	1,1	1,2	1,07	0,548	0,85–1,35
5	Malnutrition (E40-E46)	238	6,4	1,0	0,9	1,0	1,02	0,893	0,79–1,31
6	Anaemia (D55-D59)	186	5,0	0,8	0,7	0,8	1,12	0,426	0,84–1,50
7	Inflammatory diseases of the central nervous system (G00-G09)	129	3,5	0,5	0,5	0,5	1,02	0,896	0,73–1,45
8	Respiratory and cardiovascular perinatal disease (P20-P29)	103	2,8	0,5	0,3	0,4	1,63	0,019	1,08–2,47
9	Fetus and newborn affected by mother infection (P00-P04)	88	2,4	0,4	0,3	0,3	1,01	0,967	0,66–1,54
10	Infection specific to the perinatal period (Sepsis perinatal) (P35-P39)	87	2,4	0,4	0,3	0,3	1,14	0,550	0,74–1,76
11	Other bacterial disease (A30-A49)	86	2,3	0,4	0,3	0,3	1,33	0,199	0,86–2,05
12	Tuberculosis (A15-A19)	64	1,7	0,3	0,2	0,3	1,44	0,154	0,87–2,36
13	Disorders related to length of gestation and fetal growth (P05-P08)	61	1,7	0,3	0,2	0,2	1,59	0,076	0,95–2,67
14	Metabolic disorders (E70-E90)	46	1,2	0,2	0,2	0,2	0,90	0,711	0,50–1,60
15	Burns (T20-T31)	37	1,0	0,2	0,1	0,2	1,44	0,270	0,75–2,78
16	Other disorders originating in perinatal period (P90-P96)	34	0,9	0,2	0,1	0,1	1,38	0,358	0,70–2,72
17	Other external causes of accident (W00-X59)	31	0,8	0,2	0,1	0,1	2,22	0,041	1,01–4,87
18	Transport accident (V01-v99)	28	0,8	0,1	0,1	0,1	1,70	0,178	0,78–3,71
19	Injuries involving multiple body region (T00-T07)	28	0,8	0,1	0,1	0,1	0,93	0,847	0,44–1,98
20	Other	204	5,5			0,8			
	Undetermined and Unspecific symptoms	106	2,9			0,4			
	No consensus	370	10,0			1,5			

	**Total of diagnosis**	**3696**	**100**	**12,8**	**11,4**	**14,8**	**1,17**	**0,000**	**1,10–1,25**

Respiratory and cardiovascular disorders specific to the perinatal period accounted for 54.4 deaths/1000 pyrs and was the leading neonatal cause in children ≤ 28 days (data not shown). Foetus and newborn diseases affected by maternal related factors (complications of pregnancy, labour and delivery) were responsible for 51.4 deaths/1000 pyrs. Perinatal sepsis was the third principal cause of neonatal deaths with 47.1 deaths/1000 pyrs.

The mortality rate ratio for males compared to females, after controlling for age, during the study period was 1.17, (95% CI 1.10 – 1.25%; p < 0.001). This rate ratio was, in general, higher for males than for females in all diagnoses except HIV/AIDS, metabolic diseases and superficial traumatic injuries (table 3). Deaths from external causes of accident (poisoning, falls, animal bitten, drowning and suffocation) were frequent in children male compared to female with relative risk RR = 2.2 (95% CI 1.01–4.87; p = 0.041). The same observation occurs in perinatal deaths with respiratory and cardiovascular disease RR = 1.6 (95% CI 1.08–2.47; p = 0.019)

Table 4 presents mortality rates over the entire study period in children aged more than 29 days to 15 years of age. In the age group between 29 days to 1 year, malaria (12.5 deaths/1000 pyrs), ARLI (10.6 deaths/1000 pyrs) and HIV/AIDS (6.5 deaths/1000 pyrs) accounted for 37% of infant mortality.

**Table 4:** Mortality rate in children by age group in Manhiça DSS, Mozambique 1997–2006

	**Cause of death (block-ICD 10)**	CDR * 28-1 y	CDR* 1–5 y	CDR* 5–10 y	CDR* 10–15 y
1	Malaria (B50-B54)	12,5	6,1	0,7	0,4
2	ALRI (Pneumonia) (J10-J18)	10,6	1,5	0,2	0,1
3	HIV/AIDS (B20-B24)	6,5	2,1	0,1	0,1
4	Diarrhoeal diseases (A00-A09)	4,9	2,3	0,2	0,1
5	Malnutrition (E40-E46)	3,4	2,1	0,1	0,0
6	Anaemia (D55-D59)	2,7	1,5	0,6	0,1
7	Inflamatory Central nervous System (G00-G09)	2,5	0,7	0,2	0,1
8	Other bacterial disease (A30-A49)	1,9	0,2	0,0	0,0
9	Tuberculosis (A15-A19)	1,1	0,3	0,1	0,1
10	Metabolic disorders (E70-E90)	0,5	0,4	0,0	0,0
11	Burns (T20-T31)	0,3	0,3	0,1	0,1
12	Other external causes of accident (W00-X59)	0,1	0,1	0,2	0,1
13	Transport accident (V01-v99)	0,0	0,1	0,2	0,1
14	Injuries involving multiple body region (T00-T07)	0,1	0,1	0,2	0,1

	**Total of diagnosis**	**46,9**	**17,7**	**2,8**	**1,4**

* CDR – Crude Death Rate (per 1000 persons year at risk)

Malaria was very common in children 1 to 4 years of age, with 34.4% of the total diagnoses and a mortality rate of 6.1 deaths/1000 pyrs. Malaria was followed by diarrhoea disease, HIV/AIDS and malnutrition with 2.3, 2.1 and 2.1 deaths/1000 pyrs, respectively.

**Figure 1:**
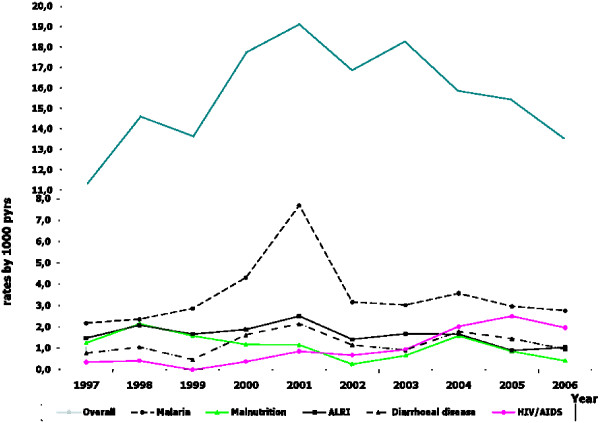
**Cause-specific death rates per 1000 person-years in children under 15 years of age, Manhiça, Mozambique**.

Figure 1 presents the cause-specific death rates during the entire study. Overall mortality increased until 2001 in the study area and then decreased during the second period between 2001 and 2006. Malaria and pneumonia largely predominated among causes of death, accounting for 30% of the mortality during the study. The first period (1997 to 2001), was marked by an increase in death rates attributed to malaria, pneumonia, diarrhoeal disease and HIV/AIDS, but not malnutrition. Deaths attributed to malaria almost triplicate between 1997 and 2001, increasing from 2.2 deaths/1000 pyrs to 7.7 deaths/1000 pyrs, respectively, and then declined substantially between 2001 and 2006 to 2.8 deaths/1000 pyrs. During the second period all main mortality causes, declined in the study area.

## Discussion

This study sought to identify the causes of death in our study area based on the verbal autopsy technique. Variability in recall period, a frequently cited limitation of VA is reported in our study. The median time was 8 months, about double that reported in other studies [14]. This is due to the data collection period used in our study that was only twice a year during the first six years of the study. However despite this limitation, interviews well recorded the signs and symptoms presented during the illness period preceding death. Other possible limitations included the relatively low specificity and sensitivity of the VA tool for detecting major causes of childhood death and the need to validate the diagnosis. In validation studies conducted in Maputo in children under 5 years old, verbal autopsy was judged to be appropriate to detect measles (sensitivities 75%, specificity 98,6%), ALRI (sensitivities, 66,7%, specificity 85,4%) and malaria (sensitivities 62,8%, specificity 90,3%) but it performed poorly for meningitis (sensitivities 33,3%, specificity 98,6%) and anaemia (sensitivities 51,9%, specificity 84,9%)[15]. These results are comparable with the results of other validation studies made in Africa, including reported by Snow et al [3], Chandramohan et al [5], Kahn et al [16] and Philip WS et al [17].

Whether to use a single or a multiple diagnostic cause is arguable particularly in the regions where patients present more than one pathology [4], as it was observed in the current study. A single diagnosis of cause of death can be used, with the assumption that other causes are ignored in the analysis. An alternative type of analysis when more than one cause of death is considered has been described by Adjuik et al [18]. He has allocated percentages of a death to the codes assigned, in proportion to the number of coders who offered a diagnosis. This methodology may underestimate individual rates per cause. For us, many times it is difficult to assign a single cause of death because the process that leads to death is complex and several diseases are involved. We try to solve this question giving the same weight for each cause of death. However it is possible that our methodology may overestimate the individual rates per cause.

In our study about 54% of all deaths took place outside a health facility. This percentage is slightly lower than that found in another study carried out in Manhiça district between 1994–96 (59,9%) [19], and is a strong reminder that access to the health facilities is not just a matter of distance, as other factors may be even more important in defining the pattern of health seeking behaviour in the area. Even when taking into account all the limitations described above, the tool may be useful in monitoring changes in mortality patterns over time.

In children under 15 years of age, mortality decreased about 30% during the last 5 years in the Manhiça study area, but the same decrease has not been observed in adults (Nhacolo A, unpublished). A main factor not only in Mozambique but in other neighbouring countries is the rapid growth of AIDS cases in adults [9].

The crude mortality rates found in this study are similar to those reported by Adjuik M, for sites as Africa Center (ACDIS) in South Africa (16.5/1000 pyrs) and Navrongo in Ghana(15.6/1000 pyrs) [18] and by Korenromp EL, for sites in Hai district (16.6/1000 pyrs) and Dar es Salam (26/1000 pyr) in Tanzania [20]. The Manhiça study area has a similar mortality pattern and high rate of deaths due to infectious disease as other African countries. The pattern of child deaths found in Manhiça is typical of developing countries [21,22].

Malaria due to *Plasmodium falciparum *was the main killer among children between 28 days to 4 years living in Manhiça area. Overall, one in four deaths was due to malaria infection. The spread of resistance to antimalarial drugs, especially chloroquine, has probably contributed substantially to this increase before 2001. After 2001, malaria cases and malaria mortality rapidly declined in the study area [23]. This may be due to several reasons such as use of more effective antimalarial drugs for treatment of non severe malaria cases such as sulfadoxine-pyrimethamine (SP) plus chloroquine initially in 2000 and SP plus amodiaquine that began in 2002. In addition, intervention studies including a malaria vaccine candidate trial for children aged 1 to 4 years, [24-26] or an the intermittent preventive treatment trial in infants using SP [27] and the distribution bed nets to pregnant mother during the last 5 years may be contributed to drop in the malaria death rate.

Pneumonia was the second overall largest cause of death in children, and was the first cause in the neonatal group. This finding is not surprising [28] and highlights the importance of some pathogens, particularly the *Streptococcus pneumoniae *and *Haemophilus Influenzae*, as major health threats to African children. The observed age pattern of pneumonia deaths, whereby neonatal infants experience the highest burden, is confirmed by morbidity data from this area [8,29]. The specific death rates decreased after 2002, particularly in children less than 5 years, when surveillance of pneumococcal disease was established.

The high ranking of HIV/AIDS in children between 29 days and 1 year explained by the growing HIV epidemic in the study area. Many HIV/AIDS deaths were probably related to malnutrition, pneumonia, malaria and diarrhoea. Given the 23.6% HIV maternal seroprevalence detected at the antenatal clinics between August 2003 and April 2005 (Menendez C – in press), we might have expected more deaths from AIDS than the reported 8.3%. It is probable that many deaths registered as diarrhoeal disease and malnutrition also had AIDS as the underlying cause, but was not reported as such. The crude death rate began decreasing later 2004, just after the implementation of antiretroviral treatment in MDH.

Diarrhoea related deaths accounted for 8% of all deaths and remains as another main contributor to child mortality in Manhiça. A similar results has been reported by Dgedge et al in children from 0 to 14 years of age in Maputo City where up to 10% of paediatric deaths are attributed to diarrhoeal [21]. In sub-Saharan Africa, hospital-based mortality from acute diarrhoea varies from 1.9% of all deaths in The Gambia to 37% in Nigeria, with most of deaths occurring during the first year of life [30]. Even though morbidity caused by diarrhoea is still high, mortality has been decreasing worldwide, also in Manhiça, mainly because of improved management and community education [31-33].

Malnutrition constitutes an important cause of child death in Africa [34]. In Manhiça the specific rate decreased during the study period due to an effective malnutrition programme in MDH that included improved detection, treatment and community follow-up at home of children after discharge from hospital. However several other factors such as poor socio-economic conditions, increasing prevalence of HIV/AIDS and tuberculosis, and the migration of the adult male population to Maputo capital and South Africa [9] may have all contributed to maintaining a high prevalence of this disease in the study area.

In Manhiça the crude mortality rate for diarrhoeal diseases, decreased at the same pace as malaria and malnutrition deaths. These related patterns suggest the relationship and possible misclassification of cause of death among these three diseases.

Finally, these results confirm that most causes of death in children are preventable. Research and programs that enable mothers to identify malaria, acute respiratory infections (particularly pneumonia) and diarrhoea, and that encourage prompt care-seeking behaviour. Strengthening case management at the primary health care facilities are important priorities. Morbidity and mortality related to prenatal causes, including asphyxia, can be reduced if staff is well-trained. Mothers should be encouraged to seek early for antepartum and intrapartum care for adequate attendance. The quality of neonatal care, with a focus on preventing infection needs to be improved.

## Conclusion

In conclusion results of this study highlight the big challenge that lies ahead in the fight against preventable infectious diseases in developing countries.

## Competing interests

The authors declare that they have no competing interests.

## Authors' contributions

JS, AQN, DN and CNS were responsible for field data collection and quality control of questionnaires. JS with BS, FA, PA, SM, TN, EVM, QB, CD, AB, EL and FS, have assigned causes of death on VA. JS, JJA and PLA were involved in the data analysis and interpretation. RT, JS and PLA participated in the design of the study and the preparation of the manuscript. JS wrote the manuscript with collaboration from all authors. All authors read and approved the final manuscript.

## Pre-publication history

The pre-publication history for this paper can be accessed here:


